# Research on Cognitive Marine Radar Based on LFM Waveform Control

**DOI:** 10.3390/s19092200

**Published:** 2019-05-13

**Authors:** Yi Liu, Shufang Zhang, Jidong Suo, Tingting Yao, Jingbo Zhang

**Affiliations:** Department of Electronic Information Engineering, Dalian Maritime University, Dalian 116026, China

**Keywords:** cognitive radar, marine radar, pulse compression radar, waveform control, environment protection radar

## Abstract

In this paper, the method of applying cognitive radar technology to marine radar is studied, and the cognitive marine radar structure and transmitted signal model with three control parameters are constructed. The selection method of waveform control parameters, which is based on the target spatial distribution and the reference target detection effect with the minimum emission energy as the criterion, is given. The transmission signal control selection method given in this paper can flexibly realize different emission signal groups of m×n×p groups by independently setting the values *m*, *n* and *p* of three control parameters. It does not require radar hardware circuit reconstruction to meet the radar waveform changes. This is more convenient for the technical realization of cognitive marine radar. According to the method of this paper, a cognitive marine radar test system was constructed. The experimental results show that the proposed radar could reduce the emission energy by 15.9 dB compared with the traditional fixed-parameter pulse compression marine radar under the experimental conditions.

## 1. Introduction

At present, marine radars always operate in a fixed parameter mode. These parameters, such as transmit pulse width and pulse repetition frequency, are only related to the working range, and are completely independent of factors such as target spatial distribution and working water characteristics. In the near-range mode, due to the narrow pulse emission, the radar range achieved is small, which makes it impossible to track and monitor targets at a long distance. When it is necessary to monitor remote targets, the radar needs to convert a large range, and it is necessary to re-establish tracking for remote targets, so that it is difficult to balance the monitoring and tracking of remote and close-in targets by the radar. For this reason, this paper studies a key technology and a method, which is to apply the technical idea of cognitive radar to marine radar, and how the method realizes cognitive marine radar. The technology and method enable the marine radar to independently control and adjust the transmitted signals and working parameters of the radar according to the working water environment and target space distribution of radar. When the radar can effectively detect and track a wide range of targets, it can also reduce the unnecessary emission of radar and reduce the radar emission energy. This not only enhances the radar work efficiency, but also reduces the radar to the environment electromagnetic radiation influence.

In 2006, Simon Haykin put forward the concept of cognitive radar [1], and clearly pointed out that cognitive function is an important symbol of new generation radar system [2]. Cognitive radar integrates brain science and artificial intelligence into radar system [2], which gives the radar system the ability to perceive the environment; understand the environment; learn, reason and judge decisions; and adapt to the trend of radar intelligence [3]. The closed-loop working structure of cognitive radar is designed to carry all available resources transmitted and received, and utilizes situational awareness of the working environment to maximize system performance and match the system to the working environment [4].

In recent years, research results on cognitive radar emerge constantly. The research focuses mainly on the design and selection of optimal transmitting waveform [5,6], optimization algorithm of resource allocation [7,8], automatic operation and management [4,9], and the spectrum sharing with communication and optimal utilization and allocation of channel resources [10,11]. A full understanding of the work environment is a fundamental feature of cognitive radar. In this way, the radar transmit waveform and working state can be matched with the radar working environment to obtain the best radar target detection performance and target state estimation performance [3,12]. At the same time, the highest radar operating efficiency and the lowest impact on the environment can be obtained [13,14]. To this end, in [15], two metrics for quantifying target tracking performance of cognitive radar are proposed, namely combined cumulative squared deviation (CCSD) and cumulative coherent processing interval (CCPI). These two metrics can be used to optimize radar signal transmission parameters to determine the pulse repetition frequency (PRF) and associated time interval that match the environment. Giusti et al. [16] analyzed a cognitive multi-platform synthetic aperture radar system design problem, and discussed a method based on cognitive and autonomous learning from observation to implement autonomous waveform diversity system to maximize its performance over time. Aittomaki et al. [17] proposed an efficient and low computational complexity method for optimizing the power allocation of MIMO radars to achieve maximum discovery probability. This method uses a simpler approximation when establishing optimization criteria for power distribution of target dynamic distribution. The distribution replaces the theoretically precise distribution. Waveform design is a constraint optimization problem with an optimal criterion, usually including one or more performance functions and constraints [18]. The choice of performance function is usually related to radar task [19,20], and different criteria describe the purpose of waveform optimization. The research on the optimal waveform design of this kind mainly focuses on the fields of MIMO radar [21,22], tracking radar [23] and so on. The above research results have played an important role in promoting the development of cognitive radar theory, technology and engineering applications. However, these techniques are not well suited for ordinary civilian radars that place too much emphasis on price and performance, such as marine radar. In the common civil application fields such as marine radar, the practical application research of cognitive radar technology has not received enough attention, and research on cognitive marine radar waveform design is still rare. Under the trend of increasing digitization of transceivers, cognitive marine radars will have more viable implementation conditions in the future. Therefore, the research in this application field also has important theoretical significance and engineering application value.

Marine radar is a radar with a transmitting and receiving common antenna, which works by tracking while scanning. The most difficult to handle situation for marine radar is sea clutter, because the background variation of sea clutter is strong and complicated. Since the non-stationary characteristics of sea clutter are complex and changeable with sea conditions and geographical environment, it is difficult to characterize sea clutter with a precise model. However, due to the slow motion of the radar carrier and the target, the sea clutter mainly manifests as non-stationarity with range and azimuth rather than with time. During the antenna scanning process, the radar can obtain a large amount of clutter observation data, thereby obtaining perceptual information about the intensity of the sea clutter at each local spatial location. At the same time, radar echo and AIS sensors can be used to obtain the distribution information of the target. It can also use the shoreline identification technology [24,25] and electronic charts to obtain water environment information. Target detection and tracking performance is mainly restricted by the signal-to-clutter ratio (SCR). Therefore, this paper controls the transmit waveform parameters based on the perception of the SCR. At a certain clutter strength, the echo energy of the target determines the SCR. Using the SCR estimation based transmit waveform parameter control method, it is possible to control the transmitted signal parameters to minimize the emission energy under the premise of ensuring the target detection performance.

The content of this paper is as follows: in Section 2, the transmitting signal model of cognitive marine radar is described, and the radar transmitting signal model is established. In Section 3, the transmission mode including a cognitive cycle and an execution cycle is established, and the principles and methods for selecting the parameters of the transmission signal during the execution cycle are given. In Section 4, the validity of the proposed method is verified by experiments in real waters and compared with the conventional method of transmitting signals with fixed parameters. The conclusions are given in Section 5.

## 2. Design of Transmitting Signal Model of Cognitive Marine Radar

Since Linear Frequency Modulated (LFM) has many advantages, it has been used to solve the several problems, for example balance and improve the relationship between the range resolution of the radar and the detection distance; solve the contradiction between distance resolution and speed resolution [26]; and effectively reduce the peak power of the emission. Therefore, this paper adopts Linear Frequency Modulated (LFM) signal as the basic form of transmitting signal.

**Figure 1:**
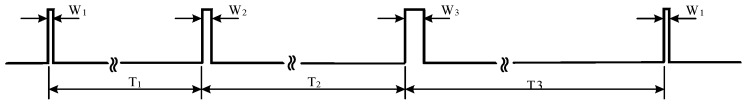
The basic waveform of the transmitted signal.

For the Track While Scan (TWS) LFM radar with the T-R common antenna, to utilize the echo signal splicing synthesis method shown in Figure 1, taking into account the minimum and maximum operating range, the transmitted signal adopts a variable parameter transmission signal in the form shown in Figure 1. The transmit waveform parameters are controlled based on the cognitive radar scene information.

In Figure 1, let the transmit pulse widths be $W_{i}=\alpha_{i}\times W_{oi}$, (i=1,2,3), which are used to achieve the radar detection on minimum range, medium range and long range, respectively, where $\alpha_{i}$ is the control coefficient; $W_{oi}$ is the basic pulse width of the *i*th pulse with fixed values, the pulse interval be $T_{i}=\alpha_{i}T_{oi}+\Delta_{i}$ (i=1,2,3), where $\Delta_{i}$ is the random time increment inserted to avoid range ambiguity. The number of achievable waveforms is determined by the number of control coefficients. Increasing the number of values $\alpha_{i}$, (i=1,2,3) increases the selectable number of waveforms. Letting the values of $\alpha_{1}$, $\alpha_{2}$ and $\alpha_{3}$ be *m*, *n*, and *p*, respectively, the number of the signal wave is $N_{w}=m\times n\times p$.

If the transmitted pulse power $P_{ti}=P_{t}$ is a constant, the transmitted signal energy is

(1) $$\begin{array}{c} E_{ti}=W_{i}P_{t}=\alpha_{i}W_{oi}P_{t} \end{array}$$

According to the energy principle of matched filter, its detection ability depends only on the signal energy. To enhance the detection capability of the detector, the control energy of the signal can be controlled by the control coefficient $\alpha_{i}$. Obviously, the signal energy transmitted by the transmitter and pulse repetition period $T_{R}=T_{1}+T_{2}+T_{3}$ is controlled by the coefficient $\alpha_{i}$. The value of $\alpha_{i}$ is adjusted by the control unit based on the radar working scene sensing information.

The echo signals generated by the three transmitted signals are pulse-compressed, time-aligned with zero-distance time as reference point, and non-coherent accumulation processing is performed, which can effectively improve the detection capability of the radar. Let the three pulse signals shown in Figure 1 form a transmission signal group, where the pulse width of the *i*th transmitting pulse is $W_{i}$, the amplitude A of the transmitted signal is constant, and the modulation bandwidth is $B_{i}$. Each group of transmitting signals is

(2) $$s\left(t\right)=\sum_{i=1}^{3}Au_{i}\left(t\right)e^{j2\pi f_{0}\left(t-\sum_{j=1}^{i}\left(j-1\right)\varepsilon_{j-1}T_{j-1}\right)}$$

here, $\left[\varepsilon_{1},\varepsilon_{2},\varepsilon_{3}\right]=\left[1,1,0.5\right]$, $u_{i}\left(t\right)$ is the normalized complex envelope of the signal, i.e.,

(3) $$u_{i}\left(t\right)=rect\left(\frac{t-\sum_{j=1}^{i}\left(j-1\right)\varepsilon_{j-1}T_{j-1}}{W_{i}}\right)e^{-j\pi K_{i}{\left(\sum_{j=1}^{i}\left(j-1\right)\varepsilon_{j-1}T_{j-1}\right)}^{2}}$$

where $K_{i}=K_{oi}/{\alpha_{i}}^{2}$ is the frequency modulation slope of the *i*th pulse of this transmitted group; $K_{oi}$ is the basic modulation slope of the *i*th pulse; $K_{oi}=0$; the modulation bandwidth of the *i*th pulse is $B_{i}=K_{oi}W_{i}/{\alpha_{i}}^{2}$; and rect(t) represents a rectangular pulse.

(4) $$rect\left(\frac{t}{W}\right)=\left\{\begin{array}{cc} 1, & |t|\leq \frac{W}{2}\\ 0, & \mathrm{else} \end{array}\right.$$

Since the amplitude A of the transmitting signal is constant, in the following analysis, to simplify the representation, it is omitted. Bringing Equation (3) into Equation (2), the transmitting signal is written as

(5) $$s\left(t\right)=\sum_{j=1}^{i}rect\left(\frac{t-\sum_{j=1}^{i}\left(j-1\right)\varepsilon_{j-2}T_{j-1}}{W_{i}}\right)\times e^{j\pi K_{i}{\left(t-\sum_{j=1}^{i}\left(j-1\right)\varepsilon_{j-2}T_{j-1}\right)}^{2}}e^{j2\pi f_{0}\left(t-\sum_{j=1}^{i}\left(j-1\right)\varepsilon_{j-2}T_{j-1}\right)}$$

**Figure 2:**
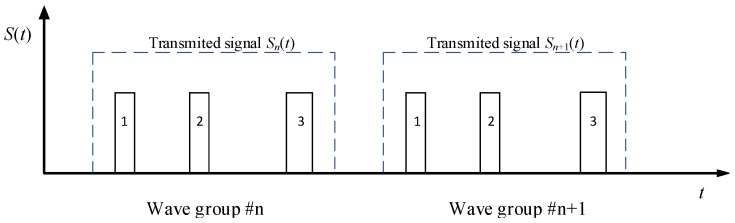
Schematic diagram of three pulses emitted by each pulse group of the radar.

Assuming that the pulse repetition period (PRP) of the radar is $T_{r}$, the radar transmits a pulse group of three pulses, as shown in Figure 2, at that PRF. In the *n*th group transmission, a combination of certain pulses under the control parameter $\alpha ={\left[\alpha_{1},\alpha_{2},\alpha_{3}\right]}^{T}$ is repeatedly transmitted *K* times. Obviously, according to the different control parameters, the pulse repetition period $T_{r}$, pulse width $W_{i},i=1,2,3$, frequency modulation bandwidth $B_{i},i=1,2,3$ and the emission energy $E=\sum_{i=1}^{3}E_{i}=\left(\alpha_{1}W_{01}+\alpha_{2}W_{02}+\alpha_{3}W_{03}\right)P_{t}$ achieved are different. From this, different pulse accumulation numbers, target detection effect, range resolution effect and precision of range measurement can be obtained.

The emission signal composed of K pulse groups shown in Figure 2 can be expressed as:

(6) $$S\left(t\right)=\sum_{K=0}^{K-1}rect\left(\frac{t-kT_{r}}{W_{p}}\right)S_{k}\left(t-kT_{r}\right)$$

among them:

(7) $$\begin{array}{c} s_{k}\left(t-kT_{r}\right)=\sum_{i=1}^{3}rect\left(\frac{t-kT_{r}-\sum_{j=1}^{i}\left(j-1\right)\varepsilon_{j-2}T_{j-1}}{W_{i}}\right)\\ \times e^{j\pi K_{i}{\left(t-kT_{r}-\sum_{j=1}^{i}\left(j-1\right)\varepsilon_{j-2}T_{j-1}\right)}^{2}}\times e^{j2\pi f_{0}\left(t-kT_{r}-\sum_{j=1}^{i}\left(j-1\right)\varepsilon_{j-2}T_{j-1}\right)} \end{array}$$

Here, $T_{r}=T_{1}+T_{2}+T_{3}$ is the repetition period of the signal group, and $W_{p}=T_{1}+T_{2}+W_{3}$ is the signal group width.

## 3. Selection Criteria and Control Algorithm for Radar Transmit Signal Parameters

This section studies and analyzes the control strategy of waveform parameters, the distance splicing method of radar echo signals, the control parameter adjustment algorithm based on reference target signal-to-noise ratio, and the working steps and processes of cognitive marine radar. In the part of waveform parameter control strategy, the relationship between the parameters of the radar transmission signal and the number of possible pulses accumulated, as well as the possible detecting distance is analyzed. It provides a basis for developing a transmission signal control parameter selection strategy. On this basis, the selection strategy of radar transmission signal control parameters is discussed and given. In the part of radar echo signal distance stitching, we analyze and discuss the radar echo signal model represented by the matrix. This model effectively reveals the relationship between the echo signals generated by the transmitting signal group constructed in Section 2. In the part of radar echo signal distance splicing, the radar echo signal model represented by the matrix is analyzed and discussed. The echo signal model in the form of the matrix reveals the method for generating the radar video echo signal conforming to the near and far. In the part of the radar waveform control process, we discuss the work content and process of the two phases of the radar scene perception and cognitive control of the transmitted signal. The content and process are based on the transmitted signal model of cognitive marine radar in Section 2.

### 3.1. Waveform Parameter Control Based on Target Spatial Distribution

The radar transmitting signal should first satisfy the target detection and surveillance. The work of radar is divided into two stages, that is, the stage of radar scene perception and the stage of transmitting signal cognitive control. In the stage of radar scene perception, the transmitted signals of the radar can realize the requirements of target detection in the radar surveillance area. In the cognitive control stage of transmitting signal, according to the target spatial distribution and water geographical environment recognized by radar scene perception stage and AIS auxiliary information, control parameters are selected to control the radar transmitting signal and make it match with the radar working environment. Let $R_{mi}^{\left(j\right)}$ and $R_{Mi}^{\left(j\right)}$ be, respectively, the minimum operating distance and maximum operating distance of the pulse generated when $\alpha_{i}\left(i=1,2,3\right)$ takes the *j*th value. *R* is the distance of the target. The selection principle of control parameters is to minimize the transmitted energy under the condition of ensuring target detection requirements.

Let the base parameters of the transmitted signal be as shown in Table 1; in this case, the signal performances of pulse-width, modulation bandwidth and time-bandwidth product achieved for Pulses 2 and 3 under various control parameters are shown in Table 2.

According to the value of the control parameters of Table 1, there are a total of 48 groups. The pulse repetition period and the maximum pulse accumulation number $M_{\max}$ at $\theta_{A}=1^{\circ }$ and $\Omega_{A}=25$ rev/min under 16 groups of different transmitting signal waveform parameters are shown in Table 3.

Table 3 shows that, by selecting $\alpha_{i}\left(i=1,2,3\right)$, 48 kinds of waveform parameters can be obtained to adapt to various target distributions. In addition, by increasing the pulse repetition rate, more pulse accumulation can be achieved, and the detection performance of the target can be improved more effectively [27]. Therefore, under the condition of certain target distribution, to ensure reliable detection of the target, pulse repetition frequency should be increased and pulse width should be reduced as much as possible to achieve greater pulse accumulation *M*. If $N_{rMi}\left(i=1,2,3\right)$ is a distance quantization unit corresponding to $R_{Mi}\left(i=1,2,3\right)$, the distance can be divided into three regions, then, according to the distance distribution of the target, the transmitting signal can be controlled according to the strategy in Table 4.

### 3.2. Waveform Control Method Based on Clutter Characteristics

The target detection processor performs target detection on the accumulated composite video signal. The accumulated echo signal can be expressed in the following two cases:

(8) $$\begin{array}{c} H_{0}:y\left(n\right)=y_{c}\left(n\right)\\ H_{1}:y\left(n\right)=y_{s}\left(n\right)+y_{c}\left(n\right) \end{array}$$

Among them, $H_{i}\left(i=0,1\right)$ is two hypothetical representations, no target existence and a target existence; $y_{s}\left(n\right)$ is a video signal generated by the target at the *n*th distance unit; and $y_{c}\left(n\right)$ is a video signal generated by sea surface reflection at the *n*th distance unit, or a noise interference term. In the radar clutter region, the impact of sea surface echo is much higher than the noise.

Assuming that the echoes generated by each range unit radar are independent of each other, obviously, according to the central limit theorem, the composite video signal shown in Equation (12), the probability density function of the clutter tends to be Gaussian distributed. That is, it can be approximated as: $H_{0}:y∼N(\mu_{H_{0}},{\sigma_{H_{0}}}^{2})$; $H_{1}:y∼N(\mu_{H_{1}},{\sigma_{H_{1}}}^{2})$, where $\mu_{H_{0}}$ and $\sigma_{H_{0}}$ are, respectively, the mean and standard deviation of the sea surface echoes in the radar illumination area; $\mu_{H_{1}}$ and $\sigma_{H_{1}}$ are, respectively, the mean and standard deviation of the target and sea clutter in the radar-irradiated area where the target exists; and the mean $\mu_{H_{1}}$ should have a linear superposition of the mean $\mu_{T}$ of the target reflection and the mean $\mu_{C}$ of the sea clutter, i.e., $\mu_{H_{1}}=\mu_{T}+\mu_{C}$.

Obviously, $\mu_{T}$ is the video mean component determined by the target reflection characteristics. $\mu_{C}$ is a stable echo component determined by sea surface reflection characteristics. If the mean value of the target cell and the adjacent clutter unit are, respectively, $\mu_{H_{1}}$ and $\mu_{H_{0}}$, according to the Gaussian distribution characteristic, then $\Delta \mu =\mu_{H_{1}}-\mu_{H_{0}}$ can be approximated to the amplitude of the target video signal, so the target echo energy can be considered as $\Delta \mu^{2}$. $\sigma_{H_{1}}$ is the standard deviation of the random variation in the target existence area. Similarly, in the radar illumination area where the target exists, the energy of the clutter is $\sigma_{H_{1}}^{2}$, and the signal-to-clutter ratio (SCR) in the target existence area can be written as:

(9) $$\mathrm{SCR}=\frac{\Delta \mu^{2}}{\sigma_{H_{1}}^{2}}=\frac{{\left(\mu_{H_{1}}-\mu_{H_{0}}\right)}^{2}}{\sigma_{H_{1}}^{2}}$$

using the processor in [28], which averages processing with respect to adaptive neighboring cells. $\mu_{H_{0}}$ and $\mu_{H_{1}}$ can be estimated by the following equations in the non-target existing area and the target existing area adjacent to the reference target.

(10) $${\hat{\mu }}_{H_{0}}=\frac{1}{2N}\left[\sum_{n=R_{\mathrm{TK}0}-N}^{R_{\mathrm{TK}0}-1}y_{H_{0}}\left(n\right)+\sum_{n=R_{\mathrm{TK}0}+L_{T}}^{R_{\mathrm{TK}0}+L_{T}+N-1}y_{H_{0}}\left(n\right)\right]$$

(11) $${\hat{\sigma }}_{H_{0}}^{2}=\frac{1}{2(N-1)}\left[\sum_{n=R_{\mathrm{TK}0}-N}^{R_{\mathrm{TK}0}-1}{\left(y_{H_{0}}\left(n\right)-{\hat{\mu }}_{H_{0}}\right)}^{2}+\sum_{n=R_{\mathrm{TK}0}+L_{T}}^{R_{\mathrm{TK}0}+L_{T}+N-1}{\left(y_{H_{0}}\left(n\right)-{\hat{\mu }}_{H_{0}}\right)}^{2}\right]$$

(12) $${\hat{\mu }}_{H_{1}}=\frac{1}{N}\sum_{n=R_{\mathrm{TK}0}+1}^{R_{\mathrm{TK}0}+L_{T}}y_{H_{1}}\left(n\right)$$

(13) $${\hat{\sigma }}_{H_{1}}^{2}=\frac{1}{N-1}\sum_{n=R_{\mathrm{TK}0}+1}^{R_{\mathrm{TK}0}+L_{T}}{\left(y_{H_{1}}\left(n\right)-{\hat{\mu }}_{H_{1}}\right)}^{2}$$

Here, $y_{H_{0}}\left(n\right)$ and $y_{H_{1}}\left(n\right)$ are video under two hypotheses, $R_{\mathrm{TK}0}$ is the target front distance unit, $L_{T}$ is the longitudinal dimension of the target expressed by the number of distance quantized units, and *N* is the length of the single-sided reference window based on the adaptive unit statistical averaging algorithm in [28]. The Probability Density Function (PDF) of the signal *y* can be expressed as:

(14) $$p\left(y|H_{i}\right)=\frac{1}{\sqrt{2\pi }\sigma_{H_{i}}}\exp\left(-\frac{{\left(y-\mu_{H_{i}}\right)}^{2}}{2{\sigma_{H_{i}}}^{2}}\right),i=0,1$$

The false alarm probability and detection probability are, respectively:

(15) $$P_{f}=\frac{1}{\sqrt{2\pi }\sigma_{H_{0}}}\int_{y_{T}}^{\infty }\exp\left(-\frac{{\left(y-\mu_{H_{0}}\right)}^{2}}{2{\sigma_{H_{0}}}^{2}}\right)dy=\frac{1}{\sqrt{\pi }}\int_{A_{f}}^{\infty }\exp\left({-r}^{2}\right)dr=\frac{1}{2}refc\left(\frac{y_{T}-\mu_{H_{0}}}{\sqrt{2}\sigma_{H_{0}}}\right)$$

(16) $$P_{d}=\frac{1}{\sqrt{2\pi }\sigma_{H_{1}}}\int_{y_{T}}^{\infty }\exp\left(-\frac{{\left(y-\mu_{H_{1}}\right)}^{2}}{2{\sigma_{H_{1}}}^{2}}\right)dy=\frac{1}{\sqrt{\pi }}\int_{A_{d}}^{\infty }\exp\left({-\xi }^{2}\right)d\xi =\frac{1}{2}refc\left(\frac{y_{T}-\mu_{H_{1}}}{\sqrt{2}\sigma_{H_{1}}}\right)$$

Obviously, by Equation (15) given the required false alarm probability, the threshold can be solved.

(17) $$\frac{y_{T}-\mu_{H_{0}}}{\sqrt{2}\sigma_{H_{0}}}=A_{f}$$

Under the false alarm probability determined by the threshold of Equation (17), given the probability of discovery required by Equation (16), the available detection threshold is

(18) $$\frac{y_{T}-\mu_{H_{1}}}{\sqrt{2}\sigma_{H_{1}}}=A_{d}$$

The detection thresholds $y_{T}$ in Equations (17) and (18) are the same, so there is the following distinguishing relation for cognition and control:

(19) $$\sqrt{2}\sigma_{H_{0}}A_{f}+\mu_{H_{0}}=\sqrt{2}\sigma_{H_{1}}A_{d}+\mu_{H_{1}}$$

where $A_{f}$ and $A_{d}$ are uniquely determined by the false alarm probability and the detection probability, respectively. Bringing Equation (9) into Equation (19), SCR can be further expressed by $A_{d}$ and $A_{f}$, as shown in Equation (20):

(20) $$SCR=2{\left(\frac{\sigma_{H_{0}}}{\sigma_{H_{1}}}A_{f}-A_{d}\right)}^{2}$$

Obviously, when $P_{f}$ and $P_{d}$ are determined, SCR varies with $\sigma_{H_{0}}/\sigma_{H_{1}}$. Let the cognitive parameters corresponding to the *n*th waveform parameter adjustment cycle be expressed as Equation (21).

(21) $$SCR\left(n\right)=2{\left(\frac{\sigma_{H_{0}}\left(n\right)}{\sigma_{H_{1}}\left(n\right)}A_{f}-A_{d}\right)}^{2}$$

The control actuator can obtain the following relationship according to Equation (21) under the condition of a certain false alarm probability and discovery probability according to the current data recorded in the working memory.

(22) $$\begin{array}{c} \begin{array}{c} \mathrm{If},\;SCR(n)<SCR(n-1),\;\mathrm{adjust}\;\mathrm{control}\;\mathrm{parameters}\;\mathrm{up}\\ \mathrm{If},\;SCR(n)>SCR(n-1),\;\mathrm{adjust}\;\mathrm{control}\;\mathrm{parameters}\;\mathrm{down}\\ \mathrm{If},\;SCR(n)=SCR(n-1),\;\mathrm{maintain}\;\mathrm{control}\;\mathrm{parameters}\;\mathrm{unchanged} \end{array} \end{array}$$

In practical use, the current SCR(n) can be obtained by substituting the estimated values ${\hat{\mu }}_{H_{1}}$, ${\hat{\mu }}_{H_{0}}$, ${\hat{\sigma }}_{H_{1}}$, and ${\hat{\sigma }}_{H_{0}}$ into Equations (21) and (9). Table 5 illustrates the relationship between $A_{f}$, $A_{d}$ and $P_{f}$, $P_{d}$, under the condition of Gaussian distribution.

### 3.3. Radar Echo Signal Distance Splicing and Composite Video Generation

**Figure 3:**
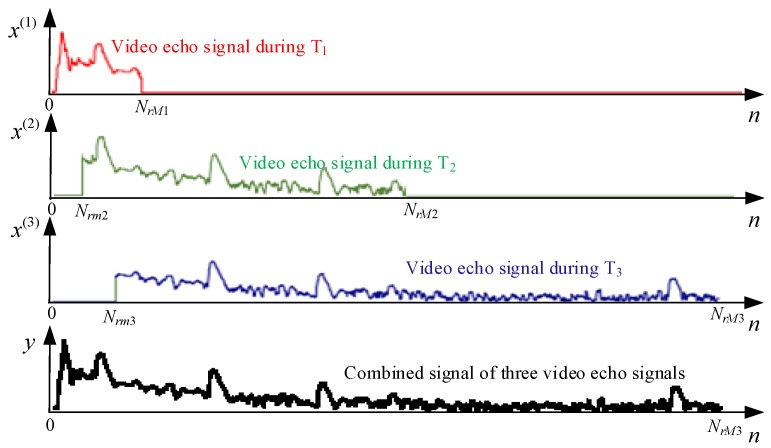
Echo video signal of the transmitted pulse group and composite video signal after the distance stitching.

Let the complex radar echo signal generated by the *n*th group of firing pulses be represented as $x_{n}\left[m,i\right]$, where *m* is the distance represented by number of distance quantization units, *i* is the transmitted pulse sequence number, the distance detection range of pulse i(i=1,2,3) is $N_{rmi}-N_{rMi},\left(i=1,2,3\right)$, and the echo signal matrix after pulse compression and time side lobe suppression is:

(23) $$X_{n}=\left[x^{\left(1\right)}x^{\left(2\right)}x^{\left(3\right)}\right]=\left[\begin{array}{ccc} x_{n}\left[0,1\right] & 0 & 0\\ x_{n}\left[1,1\right] & 0 & 0\\ \vdots & \vdots & \vdots \\ x_{n}\left[N_{rm2},1\right] & x_{n}\left[N_{rm2},2\right] & 0\\ \vdots & \vdots & \vdots \\ x_{n}\left[N_{rm3},1\right] & x_{n}\left[N_{rm3},2\right] & x_{n}\left[N_{rm3},3\right]\\ \vdots & \vdots & \vdots \\ x_{n}\left[N_{rM1},1\right] & x_{n}\left[N_{rM1},2\right] & x_{n}\left[N_{rM1},3\right]\\ 0 & x_{n}\left[N_{rM1}+1,2\right] & x_{n}\left[N_{rM1}+1,3\right]\\ \vdots & \vdots & \vdots \\ 0 & x_{n}\left[N_{rM2},2\right] & x_{n}\left[N_{rM2},3\right]\\ 0 & 0 & x_{n}\left[N_{rM2}+1,3\right]\\ \vdots & \vdots & \vdots \\ 0 & 0 & x_{n}\left[N_{rM3},3\right] \end{array}\right]$$

where $x^{\left(i\right)},i=1,2,3$ is the $N_{rM3}$ dimension vector, which is the maximum distance unit number. It can be seen from Equation (23) that the distance range can be divided into the following five distance segments (represented by distance quantization unit), and the corresponding range segments are: $0∼N_{rm2}-1$, $N_{rm2}∼N_{rm3}-1$, $N_{rm3}∼N_{rM1}$, $N_{rM1}+1∼N_{rM2}$ and $N_{rM2}+1∼N_{rM3}$, respectively. The rows in Equation (23) are summed and averaged to obtain a composite video signal during a transmit pulse group as shown in Figure 3.

(24) $${\tilde{x}}_{n}=U*\left[\sum_{i=1}^{3}x_{n}^{\left(i\right)}\right]$$

where “∗” represents the Hadamard product of the vector, and *U* is a coefficient vector determined by the distance, corresponding to the above five distance segments, and the elements of each segment are 1, 1/2, 1/3, 1/2, and 1. Let the number of pulse group accumulations be *M*, and accumulate the average of the synthesized video of Equation (24) *M* times:

(25) $$Y=\frac{1}{M}\sum_{i=1}^{M}{\tilde{x}}_{i}$$

### 3.4. Radar Waveform Control Process

The basic flow of the control of the transmitting waveform parameters for the cognitive marine radar based on LFM waveform is as follows:

#### 3.4.1. Radar Scene Perception Steps

The radar scene perception phase is divided into five steps:

Step 1: In the continuous sense detection of 2–5 radar antenna scanning cycle, the records of perceptual memory are generated. In the perceptual detection phase, the control parameters of the radar transmitting signal are: $\left[\alpha_{1}\alpha_{2}\alpha_{3}\right]=\left[111\right]$. The control parameters are stored in the execution memory of control execution unit.

Step 2: However, for systems with Geographic Information System (GIS) information, in the target detection processing unit, land shielding technology is used to mask radar echo signals in terrestrial and non-radar detection areas. In addition, the radar target detection data are fused with the location and size information of the Automatic Identification System (AIS) target to achieve target detection and generate target detection record data.

Step 3: The spatial distribution of the target is analyzed, and the estimation data of position for the nearest target and farthest target and clutter parameter are generated.

Step 4: The information record of the perceptual memory is updated, and this updated information record is then analyzed and processed to update the working memory.

Step 5: Ending the sensing detection in this perceptual phase, and transferring to the cognitive control stage for transmitting signal.

#### 3.4.2. Cognitive Control Steps of the Transmitting Signal

The cognitive control stage of the transmitting signal is divided into seven steps:

Step 1: Read information and previous control parameters $\alpha^{\left(n-1\right)}$ from the working memory and the control execution memory.

Step 2: Determine the control parameters according to Table 2.

Step 3: According to the determined control parameter, the waveform control parameter is sent to the multi-pulse echo combination and the accumulator to calculate the achievable pulse accumulation number M, and the M pulse groups form a group for continuous transmission.

Step 4: Read the perceptual memory unit after transmitting the second group and check the detection effect according to Equation (20), that is, whether the probability of detection meets the requirements. Adjust control parameters according to Equation (22) and Table 3, and store the adjusted parameters in the memory of the control execution unit.

Step 5: Repeat the process of Steps 3 and 4 within the range of azimuth determined by the working memory increment.

Step 6: Add an address offset 11 to the current address of the working memory, and repeatc the process of Steps 1–5 until the cognitive control of the antenna azimuth scanning period is completed.

Step 7: Continue the process of Steps 1–6 in this stage for *P* antenna azimuth scanning cycles (*P* can be determined according to the target motion situation and the target distance, and *P* should be appropriately reduced for fast moving targets in close range), and return to the steps of radar scene perception.

## 4. Experiment and Analysis

This section presents two experiments, one is the statistical analysis of accumulated average 54♯IPIX radar clutter data, the other is the actual radar experiment in an inland river using the cognitive marine radar experimental prototype constructed by this method. The purpose of the first experiment was to justify the assumptions underlying Section 3.4. The second experiment was to verify the effectiveness and feasibility of technical implementation of this method.

### 4.1. System Structure Model of Cognitive Marine Radar

**Figure 4:**
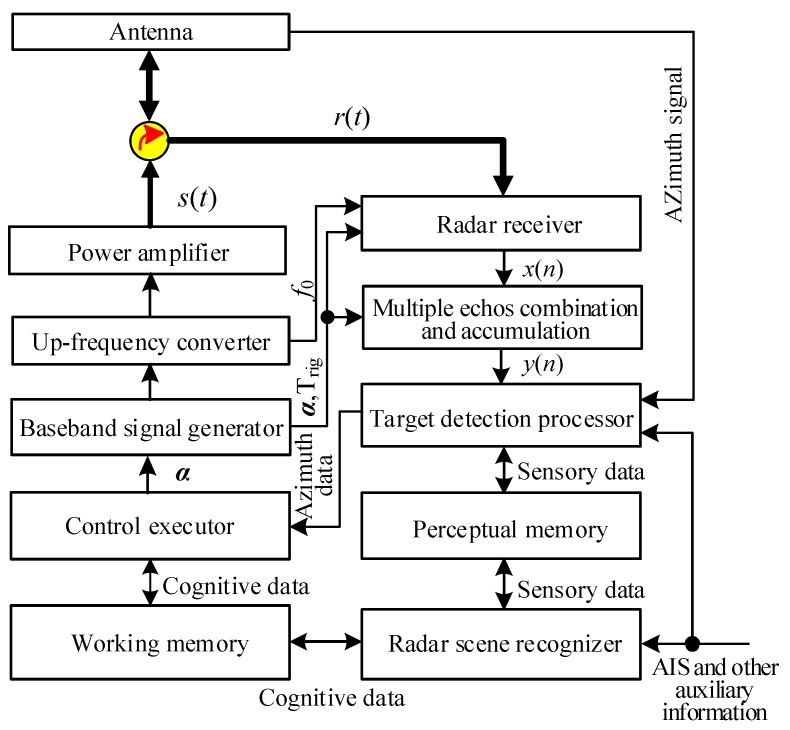
Structure block diagram of cognitive marine radar.

Stinco et al. [29] pointed out that the application of cognitive radar technology in active radar has some technical difficulties, such as cognitive requirements waveforms and real-time reconfigurability of circuits. Based on such design ideas and existing problems, this paper designs a control method based on the effective LFM waveform of marine radar. The method uses three control parameters to control the radar pulse width (energy), the transmit pulse repetition frequency and the received signal pulse accumulation number. This effectively avoids the technical problem that the signal change depends on the hardware circuit structure change. According to the transmitting signal model and waveform control parameter selection method given in the first two sections of this paper, as well as the accumulation and processing method of echo signal, and the basic architecture of cognitive radars described in [4,13], this paper presents the basic structure of cognitive marine radar as shown in Figure 4.

**Figure 5:**
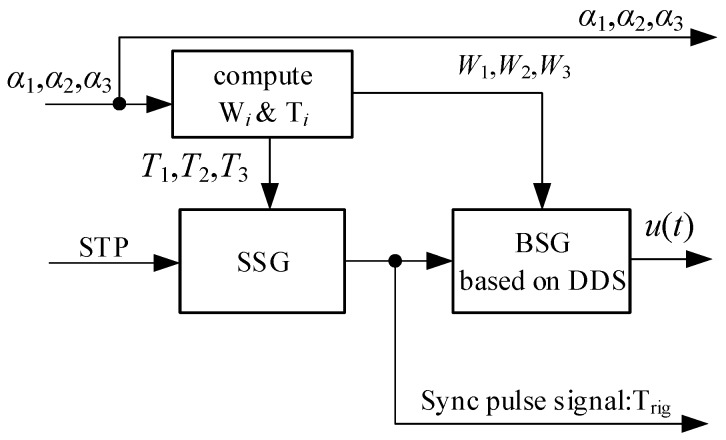
Baseband signal generator block diagram.

In Figure 4, the radar scene sensor senses the information of the target spatial distribution characteristics, the radar geographical environment, and the clutter according to the target detection status of the radar target detection unit, auxiliary information such as Automatic Identification System (AIS) and electronic chart, and generates perceptual information and cognitive information. These two kinds of information are recorded in the sensing memory and the control working memory, respectively. The radar control execution unit acquires the target spatial distribution information (cognitive data) of the target existence orientation by the working memory, and analyzes and generates the waveform control parameter $\alpha =[\alpha_{1},\alpha_{2},\alpha_{3}]$ according to the waveform selection criterion, and controls the baseband signal generator to generate the required transmit baseband signal u(t). The basic structure of the baseband signal generator is shown in Figure 5, where STP is the System Timing Pulse; SSG is the Synchronization Signal Generator; and BSG is the Baseband Signal Generator. The transmit signal waveform depends only on the control parameters and does not require hardware circuit reconstruction. The radar echo signal received by the radar is sent to the accumulator after being pulse-compressed, and its function is the combination and accumulation of multi-pulse echoes. The accumulator completes the distance splicing and accumulation of the echo signals of one of the transmitted pulse groups as shown in Figure 3, and produces a composite video signal. Then, according to the achievable pulse accumulation number, the composite video signal is subjected to amplitude accumulation, and the accumulated echo video signal is sent to the target detector for target detection processing. The radar target detector performs analysis and processing on the radar target spatial distribution characteristics, radar clutter statistical characteristics and terrestrial regional distribution characteristics while performing target detection. It stores this information in the sensing memory, and updates the working memory according to the azimuth scanning period.

The spatial distribution information of targets, the geographic environment information of radar working waters such as shoreline, and the estimation information of radar clutter intensity are obtained in the cognitive control phase composed of *P* antenna azimuth scanning cycles, by means of radar target detection, shoreline detection, radar video shoreline shielding based on electronic chart and received AIS information. The information is indexed by the azimuth, recorded in the perceptual memory in real time. Delayed by one antenna scanning period, the information such as the target distribution, etc. on the target existence orientation is transferred to the working memory. The control actuator acquires the cognitive information of the radar from the working memory with the azimuth as the index, determines the radar transmitting signal parameters corresponding to the orientation according to the waveform parameter selecting control strategy, and controls the baseband signal generator to generate a transmit baseband signal. The recorded information content of the work memory is reported as follows: The starting orientation θ of the target exists; the azimuth range Δθ of the target continuously exists; the closest range $R_{\mathrm{Tmin}}$ of the target exists; the farthest target range is $R_{\mathrm{Tmax}}$; reference target range $R_{\mathrm{TK}}$; the signal-to-clutter ratio SCR at the reference target; and reference point clutter distribution parameter estimates $\nu ={\hat{\sigma }}_{H_{0}}/{\hat{\sigma }}_{H_{1}}$ and $\mu ={\hat{\mu }}_{H_{0}}$.

### 4.2. Experimental Analysis of the Statistical Characteristics of Clutter after Accumulation

**Figure 6:**
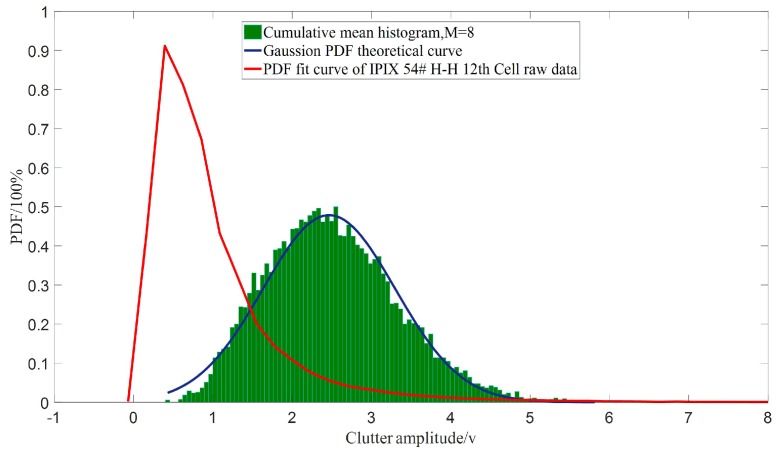
Statistical characteristics of 54♯ IPIX radar clutter before and after the accumulation and average processing.

In total, 10,000 average data were obtained by accumulation of every eight data for the 12th range unit in 54♯IPIX radar with polarization of H-H, statistically analyzed to get statistical histogram and used to estimate the mean and variance. The comparison between the distribution obtained from the histogram of the experimental data and the theoretical Gaussian distribution with mean and variance derived from experimental data is shown in Figure 6.

Figure 6 shows that the statistical distribution of radar clutter, which is similar to K distribution, is close to Gaussian distribution after accumulation and average processing with eight data of clutter. It is shown that the assumptions in Section 3.2 are credible.

### 4.3. Experimental Analysis of Statistical Characteristics of Accumulated Clutter

**Figure 7:**
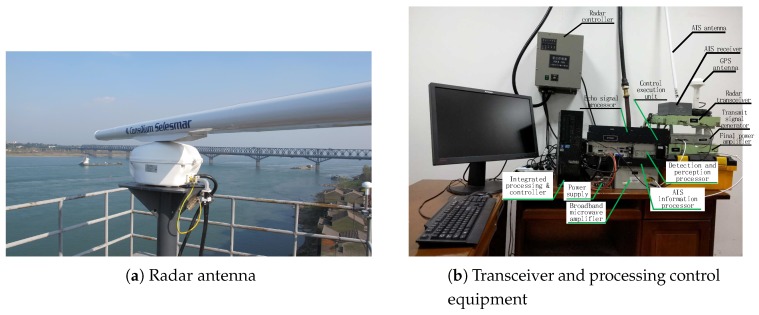
Radar experimental system experimental test site.

The field experimental system constructed according to Figure 4 is shown in Figure 7. The main radar operating parameters and main control parameters are shown in Table 6 and Table 7, respectively.

$A_{f}=4.7534243\left(P_{f}=10^{-6}\right)$, $A_{d}=-0.84162123\left(P_{d}=0.8\right)$.

The experimental site was Zhicheng Bridge radar station, the antenna height of which is about 25 m relative to the water surface, as shown in Figure 7a. Figure 7b shows the transceiver and processing equipment, including RF signal generation, microwave power amplification, echo signal processing, target detection and sensing processing, control execution processing, information processing and control, AIS receiver, AIS information processor, etc. that constituted the experimental system. In the experiment, the weather was good and the water surface was calm. The transmitted signal pulse power was 30 W.

### 4.4. Field Experiment Results and Analysis of Experimental Results

**Figure 8:**
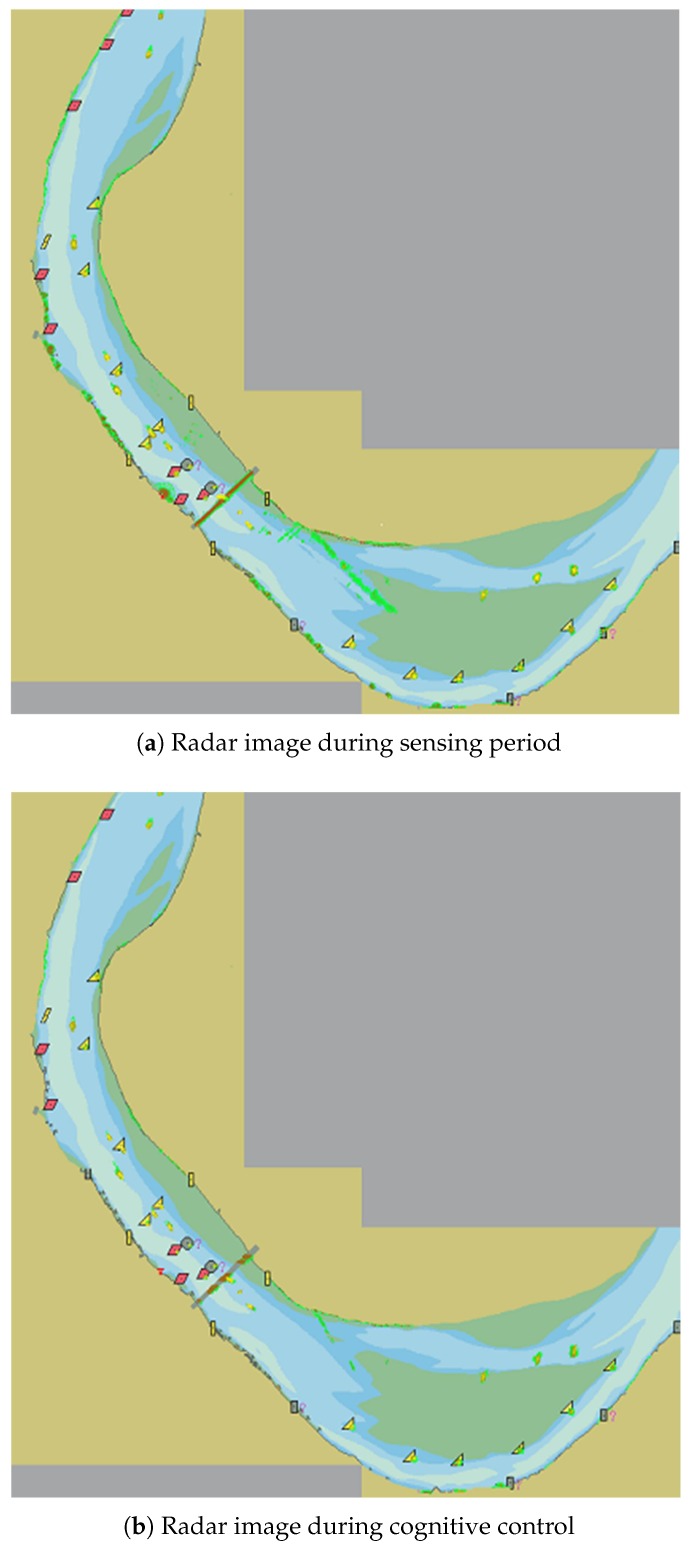
Comparison of radar images during perception and cognitive control.

The experimental system alternated between the two phases of radar scene perception and transmitted signal cognitive control in accordance with the workflow of Section 3.4. In the radar scene sensing stage, after receiving processing, the radar image during perception and cognitive control shown in Figure 8a was obtained. It can be seen from the figure that the radar video was omnidirectional and the echo was strong, because the working mode of the radar at this stage was all-round detection, and the radar transmited with basic pulse parameters. In the figure, since the terrestrial video was shielded, although the terrestrial area had a launch, there was no echo display. Obviously, at this stage, the radar transmitted with large energy for omnidirectional detection, the system could effectively realize the perception of the radar working scene, and thus realized more accurate recognition of the radar environment. In addition, the radar target tracking data and AIS target information established at this stage could be used to verify the detection effect of the target in the cognitive control phase of the transmitted signal, thereby supplementing the sensing working memory and adjusting the selection of the control parameters. This feedback adjustment made the radar’s operating characteristics more closely match the radar’s working environment.

**Figure 9:**
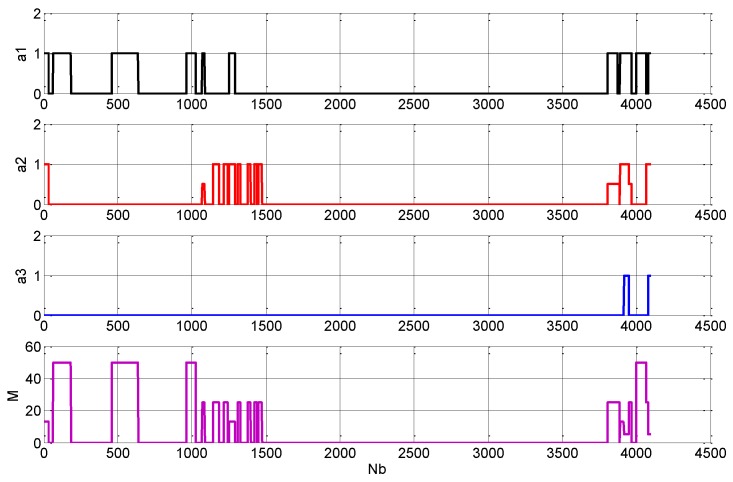
Schematic diagram of three pulses emitted by each fire group of the radar.

In the stage of the cognitive control of the transmitted signal, thd system controlled the radar transmitter to transmit energy that matched the target distance within the azimuth range in which the target existed. After the reception processing, the radar image shown in Figure 8b was obtained. As can be seen from the figure, the radar was not uniformly emitted in all directions. Compared with Figure 8a, the targets were effectively displayed. However, in the position where the target did not exist, there was no radar echo video display. This shows that the radar did not transmit signal in the direction in which the target did not exist. In contrast to Figure 8a,b also shows that the target echo intensity at a longer distance was reduced. This indicated that, in the orientation of these targets, the transmitted signal energy was lower than the transmitted energy of the radar scene sensing stage, and the radar transmission parameters were adjusted. However, the result of this adjustment still allowed the target to be effectively detected. In the cognitive control stage of the transmitted signal, corresponding to Figure 8b, the integrated processing and the controller recorded and counted the transmitted signal control parameters. Figure 9 shows the change of cognitive control parameters with azimuth of the antenna during azimuth scanning period (in the figure, Nb is the orientation expressed by the number of azimuth quantization units, and the azimuth quantization unit is $0.088^{\circ }$).

As shown in Figure 9, during cognitive control, the pulse emission only appeared in the orientation where the target existed. This is consistent with Figure 8b. Therefore, relative to the omnidirectional fixed parameter launch, the emission azimuth was only 24% of all directions. Among them, the single pulse azimuth of the pulse width $W_{01}$ accounted for 49.0%, the combined pulse of the $W_{01}$ and $0.5W_{02}$ accounted for 13.2%, the combined pulse of $W_{01}$ and $W_{02}$ accounted for 11.0%, a single pulse with $0.5W_{02}$ accounted for 1.2%, a single pulse with $W_{02}$ accounted for 21.6%, and the combined pulses of the $W_{01}$, $W_{02}$ and $W_{03}$ accounted for 4.0%. It can be seen that, in this case, the large energy emission only accounted for 4%, and the emission energy was greatly reduced. It was only 2.57% of the omnidirectional fixed parameter emission energy, i.e., it was reduced by 15.9 dB.

## 5. Conclusiosns

This paper proposes an implementation method of cognitive marine radar based on LFM waveform control, which is used to achieve a good match between radar and working environment. It discusses the structure and transmitted signal model of the cognitive marine radar, and gives the acquisition and recording method of cognitive information for the effective reduction of the radar’s emission energy under the condition of target detection performance; the signal control method of cognitive marine radar controlled by three control parameters; and the workflow of the cognitive marine radar. An experimental system based on this method was developed, and the working effect of the system was verified by field experiments. The experimental results show that the proposed method is effective and feasible. The recognition and good matching of radar working environment under experimental conditions were effectively realized, and while realizing effective detection of radar targets, unnecessary launches, launching energy and electromagnetic interference to the environment were effectively reduced.

In the structure and waveform design of information recognition in this paper, the goal is to effectively reduce the transmitting energy of radar under the condition of ensuring the performance of radar detection; the optimization of target tracking accuracy and reliability has not been fully considered. In addition, the experiments presented in this paper were only carried out in a single radar working environment of inland waters, which has certain limitations. Therefore, for the research of cognitive marine radar, further theoretical, applied and experimental research work is needed in the future.

## Figures and Tables

**Table 1:** Transmission signal reference parameter setting table.

Waveform Parameter	$T_{o1}$ $\mu_{s}$	$T_{o2}$ $\mu_{s}$	$T_{o3}$ $\mu_{s}$	$W_{o1}$ $\mu_{s}$	$W_{o2}$ $\mu_{s}$	$W_{o3}$ $\mu_{s}$	$P_{t}$ *W*
Value	100	200	700	0.1	20	100	50
Controls parameter	$\alpha_{1}$	$\alpha_{2}$	$\alpha_{3}$	$\alpha_{1}$	$\alpha_{2}$	$\alpha_{3}$	
Value	0, 1, 2	0, 0.5, 1, 2	0, 0.5, 1, 2	0, 1	0, 0.5, 1	0, 0.5, 1, 2	

**Table 2:** The signal waveform parameters and effects implemented under various control parameters.

Parameter	i=1			i=2				i=3			
$\alpha_{i}$	0	1	2	0	0.5	1	2	0	0.5	1	2
$W_{i}$ $\left(\mu_{s}\right)$	0	0.08	0.16	0	10	20	40	0	50	100	200
$B_{i}$ $\left(MH_{z}\right)$	X	12.5	6.2	X	20	20	20	X	10	10	10
$D_{i}=W_{i}B_{i}$	0	1	1	0	200	400	800	0	500	1000	2000

**Table 3:** Relationship between emission period and waveform parameters.

$\alpha_{1}$	$\alpha_{2}$	$\alpha_{3}$	$T_{r}=1/F_{r}$	$T_{r}$ $\left(\mu_{s}\right)$	$M_{\max}$	Radar Effective Range Detection Range		
0	0	0		0	0			
⋯	⋯	⋯	⋯	⋯	⋯	⋯	⋯	⋯
0	0	1	$T_{r}=T_{03}+\Delta_{3}$	700	9		$R\geq R_{m2}$	$R\leq R_{M3}$
0	1	0	$T_{r}=T_{02}+\Delta_{2}$	200	33	$R\geq R_{m2}$	$R\leq R_{M2}$	
0	1	2	$T_{r}=T_{02}+\Delta_{2}+2T_{03}+\Delta_{3}$	1600	8	$R\geq R_{m2}$	$R\geq R_{m3}$	$R\leq R_{M3}$
1	0	0	$T_{r}=T_{1}+\Delta_{1}$	100	66	$R\leq R_{M1}$		
1	1	0	$T_{r}=T_{1}+\Delta_{1}+T_{02}+\Delta_{2}$	300	22	$R\leq R_{M1}$	$R\leq R_{M2}$	
2	0.5	0.5	$T_{r}=2T_{1}+\Delta_{1}+0.5T_{02}+\Delta_{2}+0.5T_{03}+\Delta_{2}$	650	20	$R\geq 2R_{m1}$		$R\leq R_{M3}$
2	1	2	$T_{r}=2T_{1}+\Delta_{1}+T_{02}+\Delta_{2}+2T_{03}+\Delta_{3}$	1800	6	$R\geq 2R_{m1}$		$R\leq R_{M3}$
2	2	2	$T_{r}=2T_{1}+\Delta_{1}+2T_{02}+\Delta_{2}+2T_{03}+\Delta_{3}$	2000	6	$R\geq 2R_{m1}$		$R\leq R_{M3}$
⋯	⋯	⋯	⋯	⋯	⋯	⋯	⋯	⋯

**Table 4:** Control strategy for transmitting pulses.

Target Distance Distribution	$\alpha_{1}$	$\alpha_{2}$	$\alpha_{3}$	Conditional Description
$R\leq N_{rM1}$	1, 2	0, 0.5, 1	0	Increase $\alpha_{i},i=1,2$, if the probability of detection is low.
$N_{rM1}\leq R\leq 0.5N_{rM2}$	1	0.5, 1	0, 0.5	
$0.5N_{rM2}\leq R\leq N_{rM2}$	0	0, 1	0, 0.5	
$N_{rM2}\leq R\leq N_{rM3}$	0	1	0.5, 1	The radar cognitive detection time is 15–18 antenna azimuth scanning periods.
$N_{rM1}\leq R\leq N_{rM3}$	0	1	0.5, 1	
$0\leq R\leq N_{rM3}$	1	1	0, 0.5, 1	
Radar scene perception period	1	1	1	2∼5 antenna azimuth scan cycles.

**Table 5:** Relationship between $A_{f}$ and $P_{f}$ and $A_{d}$ and $P_{d}$, respectively.

$P_{f}$	$A_{f}$	$P_{d}$	$A_{d}$
$10^{-4}$	3.719016	0.85	−1.036433387
$10^{-5}$	4.26489078	0.90	−1.281551563
$10^{-6}$	4.7534243	0.95	−1.6448536241

**Table 6:** Radar main operating parameters.

Antenna Beamwidth	Radar Operating Frequency MHz	Antenna SPEED TRANSFER Minute	$P_{t}$ W	$T_{01}$μs	$T_{02}$μs	$T_{03}$μs	$W_{01}$μs	$W_{02}$μs	$W_{03}$μs	Chirp Rate MHz/s		
										$k_{01}$	$k_{02}$	$k_{03}$
$0.75^{\circ }$	9410	25	30	100	200	700	0.1	10	50	0	2	0.2

**Table 7:** Main control parameters.

*i*	1			2			3		
$\alpha_{i}$	0.5	1	2	0.5	1	2	0.5	1	2
$R_{mi}$ (km)	0.02	0.02	0.02	0.75	1.5	3.0	3.75	7.5	15
$R_{Mi}$ (km)	6	6	6	15	30	60	30	60	120
